# Pediatric Postoperative Acute Kidney Injury After Noncardiac Surgery: A Systematic Review and Meta‐Analysis

**DOI:** 10.1002/pan.70284

**Published:** 2026-08-18

**Authors:** Arash Emami, Ebba Sivertsson, Johan Westerbergh, Michael Marks‐Hultström, Johan Danielsson, Peter Frykholm, Robert Frithiof

**Affiliations:** ^1^ Anesthesiology and Intensive Care, Department of Surgical Sciences Uppsala University Uppsala Sweden; ^2^ Uppsala Clinical Research Centre Uppsala University Uppsala Sweden; ^3^ Department of Medical Cell Biology, Integrative Physiology Uppsala University Uppsala Sweden; ^4^ Department of Women's and Children's Health Uppsala University Uppsala Sweden

**Keywords:** acute kidney injury, KDIGO, pediatric AKI, pediatric anesthesia, pediatric surgery, postoperative AKI, postoperative complications, systematic review

## Abstract

**Background:**

Postoperative acute kidney injury (AKI) is associated with substantial morbidity and mortality. Reliable estimates of AKI after surgery in children are lacking. To address this gap, we aimed to synthesize the available evidence on the incidence of AKI after pediatric noncardiac surgery.

**Methods:**

We searched PubMed, Cochrane and Web of Science for studies reporting the incidence of AKI, defined according to the Kidney Disease: Improving Global Outcomes (KDIGO) criteria, within 7 postoperative days. Observational studies and clinical trials including patients aged < 18 years were eligible. The review followed the Preferred Reporting Items for Systematic Reviews and Meta‐Analyses (PRISMA) guidelines, and certainty of evidence was assessed using the Grading of Recommendations Assessment, Development and Evaluation (GRADE) approach.

**Results:**

Twenty‐seven studies (*n* = 21 908) were included. Considerable heterogeneity (*I* ^2^ = 98.7%) and likely overrepresentation of clinically distinct high‐risk populations limited the interpretability of a single overall pooled incidence estimate. The incidence was 22.3% (95% CI, 11.2–39.3) after neonatal surgery, 30.1% (95% CI, 21.4–40.6) after pediatric liver transplantation, and 9.2% (95% CI, 3.3–23.6) in mixed pediatric surgical populations. The certainty of evidence was rated low to very low. In mixed pediatric surgical populations, AKI incidence increased from 2.4% in American Society of Anesthesiologists (ASA) class 1–2 to 7.5% in ASA 3 (*p* < 0.001) and 18.9% in ASA 4–5 (*p* < 0.001), and decreased from 9.8% at 0–2 years to 4.0% at 12–18 years (*p* < 0.001).

**Conclusion:**

The reported incidence of postoperative AKI in children undergoing noncardiac surgery remains uncertain due to substantial heterogeneity and overrepresentation of high‐risk populations. AKI incidence was high in neonates and liver transplant recipients and was associated with younger age and higher ASA class, although data were limited. Further prospective studies in broader pediatric surgical populations are needed to better assess the risk of postoperative AKI in children.

**Trial Registration:**

PROSPERO registration no.: CRD42024518832

## 1 Introduction

Acute kidney injury (AKI) refers to a rapid decline in renal function, reflected by increased plasma creatinine concentration or decreased urine output [1]. In hospitalized children, AKI is common and associated with increased mortality [2], while survivors remain at increased risk of subsequent chronic kidney disease [3].

Postoperative AKI, defined as AKI occurring within 7 days of surgery, is a clinically significant complication in adults, associated with increased morbidity, longer ICU and hospital stays, higher mortality and a greater risk of major adverse kidney events at 90 days [4]. Therefore, postoperative AKI imposes substantial healthcare and economic burden yet remains potentially amenable to early detection and prevention [5].

While the clinical impact of postoperative AKI is well established in adult surgical populations and after pediatric cardiac surgery [5, 6], corresponding data in children after noncardiac surgery are limited. Quantifying the incidence of pediatric postoperative AKI provides a foundational epidemiological metric, important for both clinical practice and future research. Reliable estimates are critical for identifying modifiable risk factors, designing adequately powered interventional trials, and evaluating the impact of perioperative management strategies. Understanding how often AKI occurs also highlights unmet needs in monitoring, biomarker development and postoperative care pathways.

The aim of this study was to systematically review the literature and assess the incidence of AKI in the pediatric noncardiac surgical population.

## 2 Methods

### 2.1 Protocol and Registration

The protocol for this systematic review was prospectively registered in the International Prospective Register of Systematic Reviews (PROSPERO; CRD42024518832) on May 24, 2024 and has been published [7].

The review was conducted in accordance with the Preferred Reporting Items for Systematic Reviews and Meta‐Analyses (PRISMA) guidelines [8], and the completed PRISMA checklist is provided in the Supporting Information (Data S2).

Methodological procedures followed the recommendations of the Cochrane Collaboration [9].

Risk of bias was assessed using the ROBINS‐I tool for observational studies and the RoB 2.0 tool for randomized controlled trials [10, 11].

The overall certainty of evidence was evaluated using the Grading of Recommendations Assessment, Development and Evaluation (GRADE) approach [12].

### 2.2 Eligibility Criteria

#### 2.2.1 Study Selection

Articles based on observational and clinical trial data reporting the incidence of postoperative AKI in pediatric patients undergoing noncardiac surgery were considered for inclusion. Studies including both cardiac and noncardiac procedures were eligible only if the noncardiac cohort was reported separately. The search was limited to studies on human populations and published in English. Case reports, case series, reviews, abstracts, editorials and letters were excluded.

#### 2.2.2 Patient Population

We included data from pediatric patients (< 18 years) undergoing noncardiac surgery, with no restrictions on surgical type other than the exclusion of cardiac procedures.

Studies including both adults and pediatric populations were considered if the data for pediatric subgroups were reported separately.

#### 2.2.3 Definition of Postoperative Acute Kidney Injury

Eligible studies were required to define postoperative AKI according to the Kidney Disease: Improving Global Outcomes (KDIGO) criteria, defined by KDIGO as AKI occurring within 7 days postoperatively [1, 4]. Studies were included if the KDIGO criteria were applied using either a documented preoperative serum creatinine value or a value calculated from a presumed estimated glomerular filtration rate [13].

Studies that used non‐KDIGO definitions of AKI or did not clearly specify a seven‐day postoperative timeframe for AKI diagnosis were excluded.

### 2.3 Information Sources and Search Strategy

A systematic literature search was conducted in the following databases: PUBMED, Cochrane Library and Web of Science.

The search strategy was developed in collaboration with a medical librarian at Uppsala University, Sweden. The literature search was constructed using a combination of Medical Subject Headings (MeSH) and Title/Abstract keywords related to pediatric postoperative acute kidney injury.

An initial search was conducted on March 6, 2024, covering studies published between March 2012 and January 2024. This start date was chosen to align with the publication of the KDIGO criteria in March 2012, as only studies applying these diagnostic criteria were eligible for inclusion. An updated search was performed on 14 February 2025, identifying studies published from January 2024 to February 2025 to ensure that the review reflects the most current evidence.

Reference lists of all included studies were screened to identify additional eligible studies. The complete search strategy is provided in the Supporting Information (Data S1).

### 2.4 Selection Process

Search results were exported to a reference management system (EndNote v.20, Clarivate, Philadelphia, PA, USA) for removal of duplicates. The remaining records were then imported into Rayyan software (Rayyan, Qatar Computing Research Institute, HBKU, Doha, Qatar) [14] for screening.

Two reviewers (A.E. and E.S.) independently and in a blinded manner screened titles and abstracts for potential eligibility. Full‐text articles of potentially relevant studies were subsequently assessed independently by the same reviewers to determine final inclusion based on the predefined criteria. Any disagreements or uncertainties regarding study eligibility or inclusion were resolved through consultation with a third reviewer (R.F.). Two corresponding authors were contacted to obtain supplementary data.

### 2.5 Data Extraction

Two reviewers (A.E. and E.S.) independently extracted data using a predefined Microsoft Excel spreadsheet. The following information was collected from each included study: author, title, year of publication, number of participants, age and gender distribution, incidence of postoperative AKI, KDIGO staging (when available), type of procedure (elective or emergency), mortality, perioperative factors associated with AKI, and the method by which KDIGO criteria were assessed postoperatively.

### 2.6 Study Outcomes

#### 2.6.1 Primary Outcome

The primary outcome was the pooled incidence of pediatric postoperative AKI, defined according to the KDIGO criteria, following noncardiac surgery.

#### 2.6.2 Secondary Outcomes

The secondary outcomes were the stratified pooled incidence of postoperative AKI across different surgical populations and the pooled incidence of the different stages of AKI within these populations. Subgroup analyses were performed to assess incidence and associations according to sex, age, body weight, American Society of Anesthesiologists (ASA) classification, the method used to apply the KDIGO criteria and duration of surgery.

### 2.7 Risk of Bias Assessment

Two reviewers (A.E. and E.S.) independently evaluated the risk of bias of included studies using the Revised Cochrane risk‐of‐bias tool for randomized trials (RoB 2) and the Risk Of Bias In Non‐randomized Studies of Interventions tool (ROBINS‐I) for non‐randomized studies [9, 10, 11]. Studies were categorized accordingly to low, moderate or serious risk of bias. Any disagreements were resolved through discussion with a third reviewer (R.F.).

The results of the risk of bias assessments are presented graphically using the *robvis* package [15].

### 2.8 Certainty Assessment

Quality of evidence was assessed by The Grading of Recommendations Assessment, Development and Evaluation (GRADE) approach and summarized in GRADE evidence profiles. Two authors (A.E. and E.S.) independently assessed outcomes in accordance with GRADE guidelines.

### 2.9 Data Analysis and Statistical Methods

All analyses were based on summary data reported in the included studies; individual patient‐level data were not available.

For each study, the incidence of AKI with 95% confidence intervals (CIs) was derived from the number of AKI cases and total sample size. Confidence intervals were calculated using the Wald method [16].

Statistical heterogeneity was evaluated using Cochran's *Q* test, with τ^2^ and the *I* ^2^ statistic reported as measures of between‐study variance and inconsistency. Given the substantial heterogeneity across studies, a random‐effects model was used to calculate the pooled incidence.

Publication bias was assessed by visual inspection of funnel plots and formal tests of asymmetry using Peters' and Egger's tests.

Due to substantial heterogeneity and pronounced physiological differences across surgical populations, a sensitivity analysis of the pooled incidence estimate was considered of limited scientific value. Instead, subgroup analyses using weighted incidence of postoperative AKI were performed.

Random‐effects models were applied for within‐group incidence estimates. Associations of age and ASA class with AKI were assessed using mixed logistic regression models.

### 2.10 Protocol Amendments

The protocol was amended to extend the search window to ensure inclusion of the most up‐to‐date evidence [7].

In the protocol, a planned outcome was stratification by surgical procedures. During data extraction this proved infeasible due to inconsistent reporting. However, the included studies grouped naturally into three surgical populations (neonatal surgery, pediatric liver transplantation and a broad general pediatric cohort including all aspects of noncardiac surgery), and our secondary outcome was adjusted accordingly.

Although the protocol prespecified that conclusions would be based on studies at low risk of bias, the KDIGO‐based eligibility criteria meant that many otherwise relevant studies were rated at higher risk due to missing baseline data. We considered these studies still to be clinically informative and included them in the meta‐analysis together with their reported risk of bias.

## 3 Results

### 3.1 Study Selection

The final search, updated on February 14, 2025, yielded 3345 records. After removal of duplicates, 2336 titles and abstracts were screened, of which 243 full‐text articles were assessed for eligibility. Thirty‐one studies met the inclusion criteria. Following risk‐of‐bias assessment, four studies were excluded owing to critical risk [17, 18, 19, 20], leaving 27 studies for inclusion (Figure 1). Reasons for exclusion are provided in the Supporting Information (Table S1).

**FIGURE 1:**
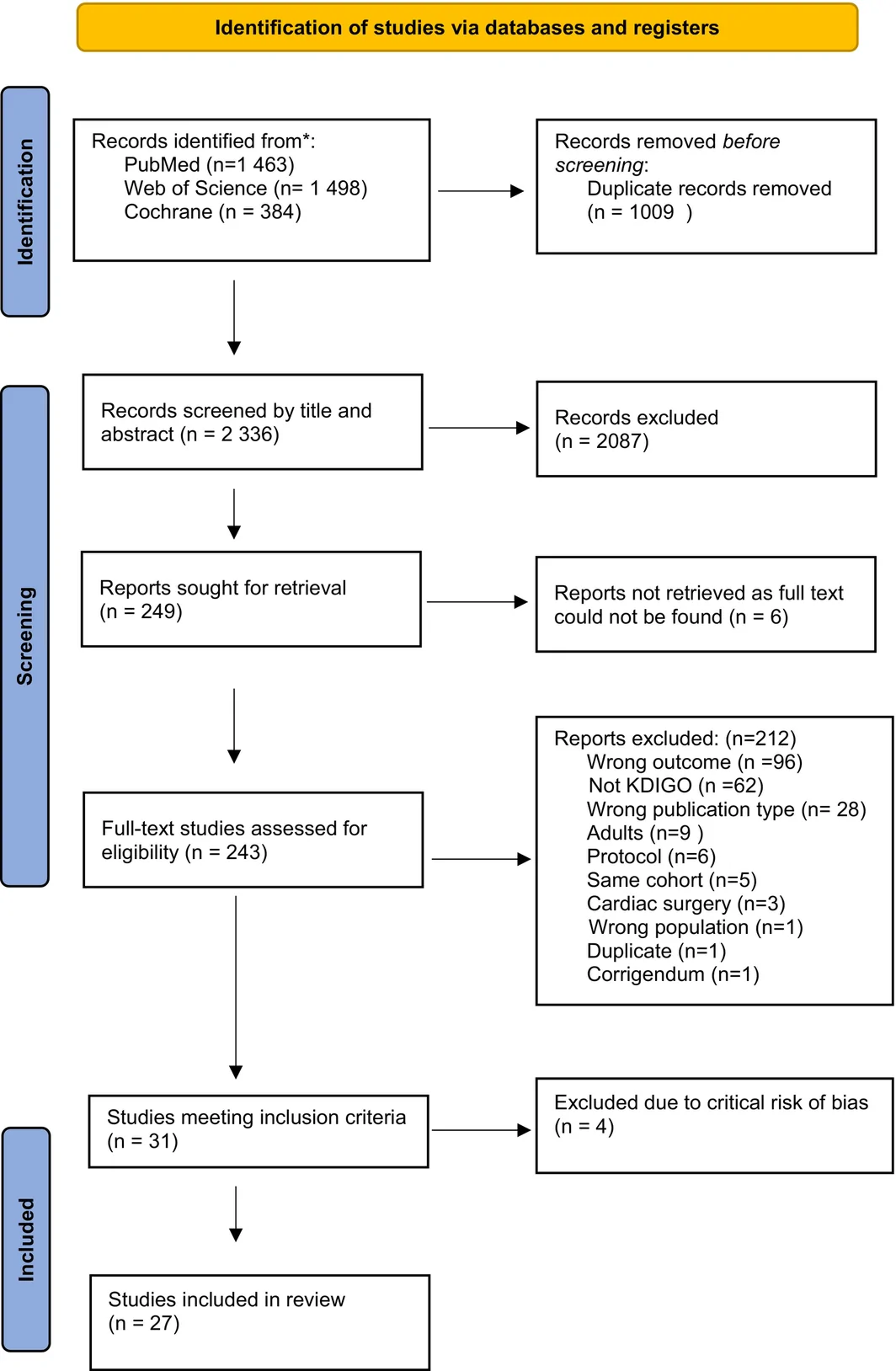
PRISMA 2020 flow chart of study inclusion, from Page et al. [6]. PRISMA, Preferred Reporting Items for Systematic Review and Meta‐Analysis Protocols.

### 3.2 Study Characteristics

The 27 included studies were published between 2017 and 2024, comprised two randomized controlled trials [21, 22], two prospective cohort studies [23, 24] and 23 retrospective cohort studies [25, 26, 27, 28, 29, 30, 31, 32, 33, 34, 35, 36, 37, 38, 39, 40, 41, 42, 43, 44, 45, 46, 47], including a total of 21 908 patients.

Of the included studies, one was multicenter and the remainder were single‐center. The studies originated from 9 countries: 1 from Africa, 7 from North America, 15 from Asia and 4 from Europe. Study characteristics are summarized in Table 1.

**TABLE 1:** Study characteristics.

Author	Year	Country	*n*	AKI Incidence %	Surgical population	Study type
Acuñas‐Soto et al. [25]	2023	Spain	181	11.6	Neonatal	SC Retro cohort
Azpilicueta et al. [26]	2023	Spain	52	28.8	Liver transplant	SC Retro cohort
Cui et al. [27]	2023	China	329	9.1	Neonatal	SC Retro cohort
Dai et al. [28]	2021	China	333	3.9	Liver transplant	SC Retro cohort
Demiroz et al. [42]	2024	Turkey	235	26.4	Liver transplant	SC Retro cohort
Fuhrman et al. [23]	2020	USA	16	37.5	Liver transplant	SC Prosp Cohort
Ha et al. [46]	2024	USA	207	16.4	Pediatric	SC Retro cohort
Hamada et al. [29]	2017	Japan	156	46.2	Liver transplant	SC Retro cohort
Ji et al. [44]	2024	China	119	45.4	Liver transplant	SC Retro cohort
Lawal et al. [24]	2019	Nigeria	93	35.5	Pediatric	SC Prosp cohort
Li et al. [21]	2019	China	120	37.5	Liver transplant	SC RCT
Liu [22]	2024	China	76	59.2	Liver transplant	SC Retro cohort
Luo et al. [43]	2024	China	4901	2.5	Pediatric	SC Retro cohort
Menon et al. [30]	2020	USA	112	38.4	Liver transplant	SC Retro cohort
Sahinturk et al. [31]	2023	Turkey	132	18.2	Liver transplant	SC Retro cohort
Schacham et al. [32]	2022	USA	4506	11.1	Pediatric	MC Retro cohort
Uasuwannakul et al. [45]	2024	Thailand	108	20.4	Liver transplant	SC Retro Cohort
Wang et al. [33]	2023	China	211	34.1	Liver transplant	SC Retro cohort
Williams et al. [34]	2017	Canada	144	67	Liver transplant	SC Retro Cohort
Wingert et al. [35]	2021	USA	8924	3.2	Pediatric	SC Retro cohort
Winters et al. [36]	2023	USA	129	24.8	Liver transplant	SC Retro cohort
Wu et al. [37]	2020	China	160	33.8	Neonatal	SC Retro cohort
Wu et al. [38]	2023	China	304	23.4	Liver transplant	SC Retro cohort
Yum et al. [39]	2019	South Korea	53	49.1	Neonatal	SC Retro cohort
Zhang et al. [47]	2024	China	106	23.6	Neonatal	SC Retro cohort
Zhang et al. [41]	2022	China	121	6.6	Liver transplant	SC RCT
Zhang et al. [40]	2020	China	80	47.5	Liver transplant	SC Retro cohort

*Note:* Characteristics of included studies. Incidence refers to the ratio of patients developing acute kidney injury based on the KDIGO‐criteria within 7 days of surgery in general anesthesia. Superscript numbers indicate references.

Abbreviations: MC, multicenter; Obs, observational; Prosp, prospective; RCT, randomized controlled trial; Retro, retrospective; SC, single‐center.

Review of the included studies identified three distinct surgical populations: neonatal surgery, pediatric liver transplantation, and mixed pediatric surgical populations. This grouping reflected a clinically meaningful separation of populations identified during the review, based on differences in patient characteristics and surgical context.

### 3.3 Risk of Bias in Studies

According to RoB 2 and ROBINS‐I assessments 11 studies were identified as having a low risk of bias, 12 as moderate, 4 as serious and 4 as critical. Studies with critical risk were excluded. The main concern across evaluated studies was missing data, most frequently owing to absent baseline creatinine measurements.

Summary distributions are shown in Figures 2a,b, and domain‐level assessments for each study presented as traffic light plots are provided in Supporting Information (Figure S1a,b).

**FIGURE 2:**
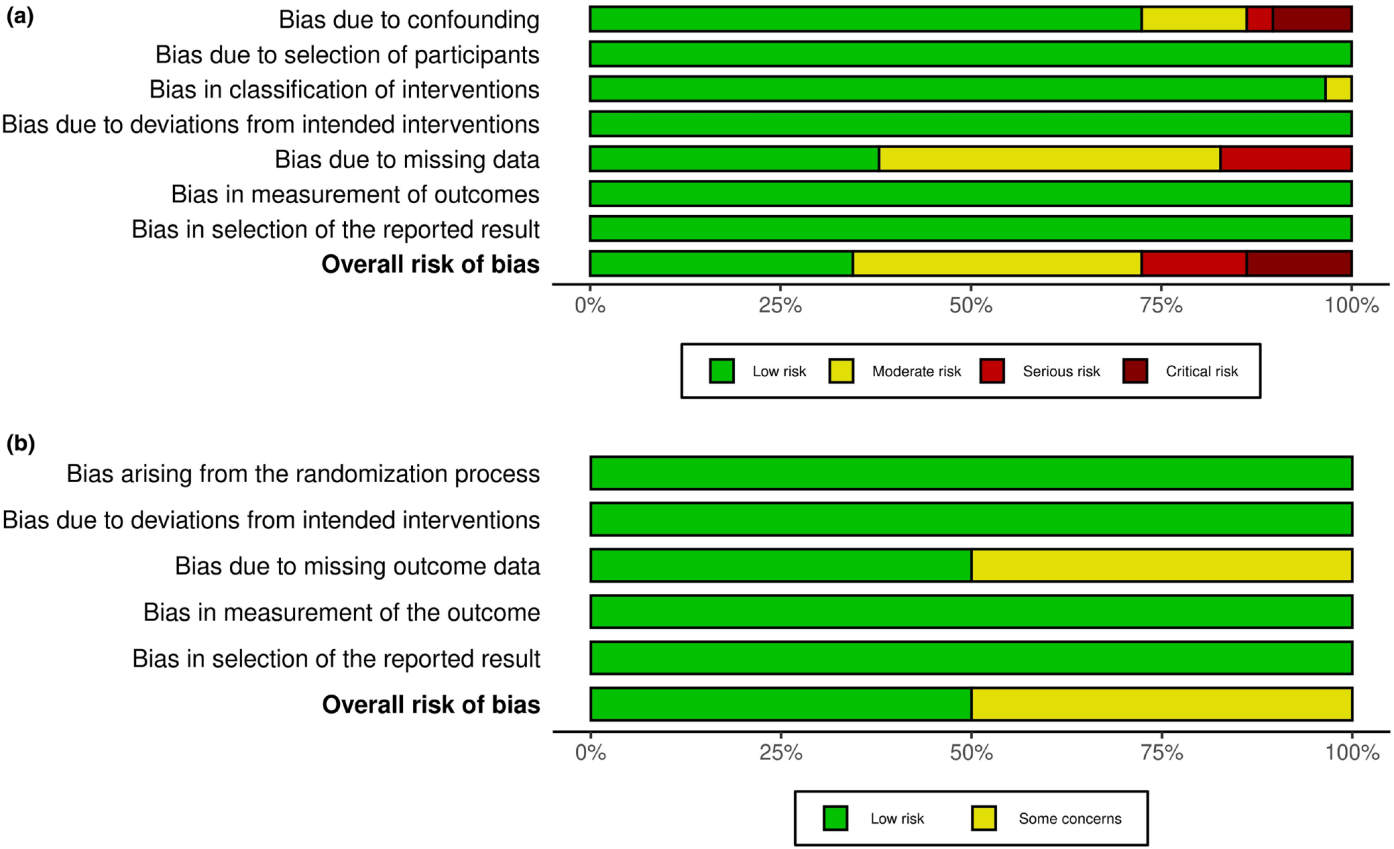
(a) ROBINS‐I risk of bias distribution for non‐randomized studies (*n* = 25), including four critically biased studies excluded from the review. (b) RoB 2 risk of bias distribution for randomized controlled trials (*n* = 2).

### 3.4 Pooled Incidence of Pediatric Postoperative AKI After Noncardiac Surgery

This meta‐analysis includes 27 studies (21 908 participants) assessing postoperative AKI defined by KDIGO criteria. Using a random‐effects model, the pooled incidence was 23.3% (95% CI: 16.5–31.8), with considerable heterogeneity across studies (*I* ^2^ = 98.7%). Figure 3 displays the overall incidence across pediatric noncardiac surgical populations, with larger studies including broader cohorts generally reporting lower rates.

**FIGURE 3:**
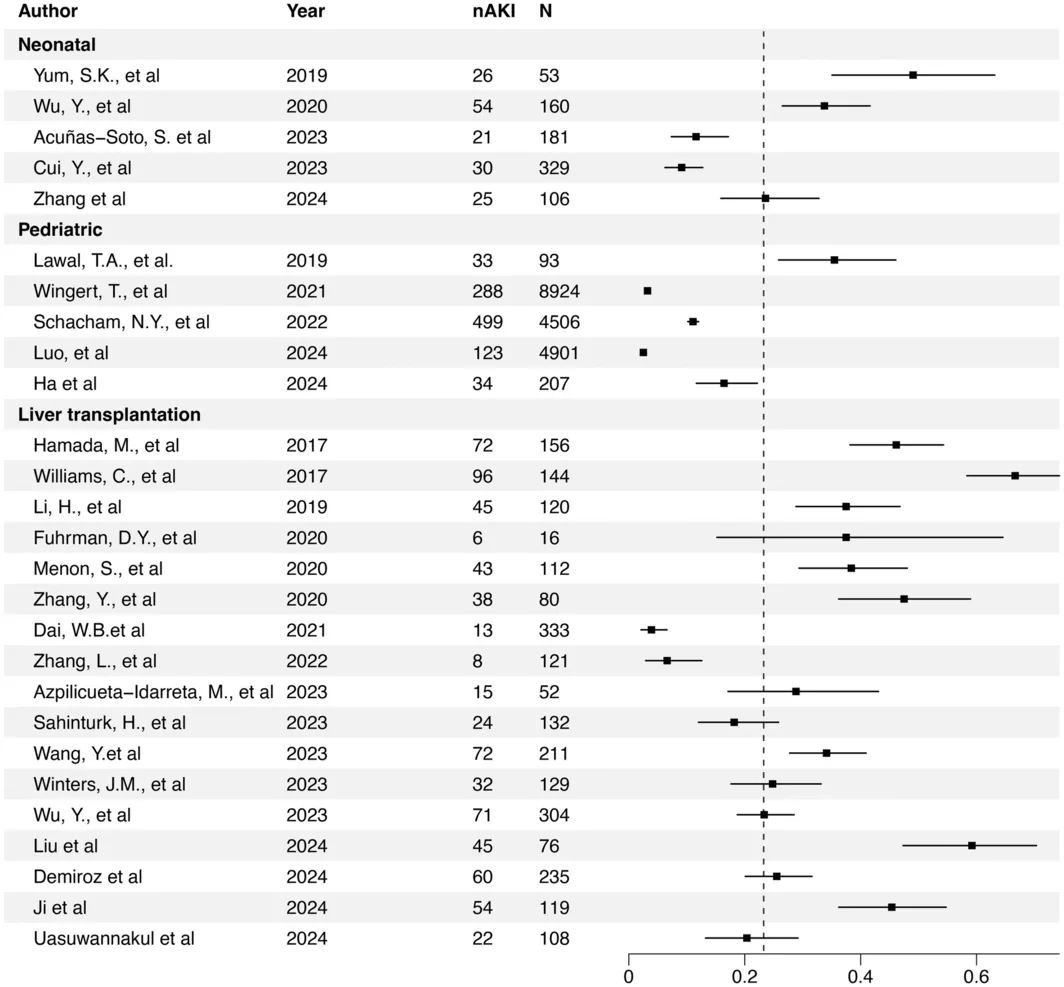
Forest plot showing the incidence rate for each study with corresponding 95% confidence intervals. The pooled incidence estimated using a random‐effects model (23.3%) is indicated by the dashed line.

### 3.5 Secondary Outcomes

Due to the pronounced between‐group differences, we did not present an overall pooled estimate for the secondary outcomes, as subgroup‐specific analyses were considered to provide a more representative summary of the data. Pooled incidences were estimated using random‐effects models, and secondary outcomes were analyzed within the predefined surgical populations.

#### 3.5.1 Incidence by Surgical Population

##### 3.5.1.1 Neonatal

In neonatal surgery (five studies, 829 participants), the pooled incidence of postoperative AKI was 22.3% (95% CI: 11.2–39.3), with substantial heterogeneity (*I* ^2^ = 94.5%). The included studies primarily involved preterm neonates, with gestational ages ranging from 27 + 2 to 37 + 2 weeks and birth weights from 1000 to 3200 g of whom 50.9% were male. Risk of bias was judged low in two studies and moderate in three, with missing data being the main concern.

##### 3.5.1.2 Liver Transplantation

In pediatric liver transplantation (17 studies, 2448 participants), the pooled incidence of postoperative AKI was 30.1% (95% CI: 21.4–40.6), with substantial heterogeneity (*I* ^2^ = 93.9%). The pooled mean age was 2.0 years, body weight 11.0 kg, and 51.6% of participants were male. Biliary atresia was by far the most common indication for liver transplantation in this cohort, followed by metabolic disease and acute liver failure.

Risk of bias was judged low in nine studies, moderate in seven and serious in one, with missing data being the main concern.

##### 3.5.1.3 Mixed Pediatric Surgical Populations

The five studies represented a broad spectrum of noncardiac pediatric surgical procedures, comprising a total of 18 631 participants. The pooled incidence of postoperative AKI was 9.2% (95% CI: 3.3–23.6), with substantial heterogeneity (*I* ^2^ = 99.3%). The pooled mean age was 8.9 years, and the overall proportion of male participants was 55.9%. Risk of bias was judged moderate in two studies and serious in three, largely because of missing baseline or postoperative creatinine values.

Surgical categories and AKI incidence varied across studies (Table 2); when available, AKI incidence in emergency surgery was reported separately.

**TABLE 2:** Surgical categories and reported AKI incidence in mixed pediatric surgical populations.

Author	Surgical categories	AKI incidence (overall)	AKI incidence (emergency surgery)
Luo et al.	Mixed (abdominal, orthopedic, neuro, thoracic, urologic)	2.5%	Not reported
Ha et al.	Urologic (lower urinary tract reconstruction)	16.4%	Not reported
Lawal et al.	General surgery (gastrointestinal, urologic, 26% preoperative sepsis)	35.5%	37.2% (no significant difference)
Wingert et al.	Mixed + procedural (cardiology, otolaryngology, gastrointestinal/hepatic, urologic, neurosurgery, orthopedic, radiology)	3.2%	6.8% (no significant difference after multivariate Cox analysis)
Schacham et al.	Mixed + procedural (categorized by organ systems)	11%	12.2% (no significant difference)

*Note:* Surgical categories and reported AKI incidence across included studies. Where available, incidence in emergency surgery is shown. Differences in definitions and reporting limit comparability between studies.

#### 3.5.2 Incidence by KDIGO Stage

##### 3.5.2.1 Neonatal

Four studies, including 776 neonates, classified AKI according to KDIGO stage. Pooled incidences were 7.3%, 3.5% and 4.0% for AKI Stage 1, 2 and 3, respectively (Table 3), with certainty levels assessed very low to low.

**TABLE 3:** Incidence per KDIGO stage.

KDIGO‐stage	No. of studies	No. of individuals	Incidence (%) (95% CI)	Certainty level
Neonatal				
Stage 1	4	776	7.3 (3.2–16.1)	Very low^a,b^
Stage 2			3.5 (1.5–8.2)	Low^b,c^
Stage 3			4.0 (0.9–16.5)	Very low^a,b^
Pediatric				
Stage 1	3	13 523	7.0 (1.5–26.3)	Very low^a,b^
Stage 2			3.2 (0.9–10.7)	Very low^a,b,c^
Stage 3			0.7 (0.2–2.3)	Very low^a,b^
Liver transplantation				
Stage 1	12	1426	15.5 (12.0–19.7)	Low^b,c^
Stage 2			13.1 (9.7–17.6)	Low^b,c^
Stage 3			5.9 (4.1–8.5)	High

*Note:* Incidence of AKI by KDIGO stage within surgical populations. Values represent pooled estimates with 95% confidence intervals. Certainty of evidence (GRADE) was downgraded for (a) Risk of bias, (b) Inconsistency and (c) Imprecision.

Abbreviation: *N* = number.

##### 3.5.2.2 Pediatric Liver Transplantation

Twelve studies, including 1426 liver transplant recipients, classified AKI according to KDIGO stage. The incidences were 15.5%, 13.1% and 5.9% for Stages 1, 2 and 3, respectively (Table 3), with certainty levels ranging from low to high.

##### 3.5.2.3 Mixed Pediatric Surgical Populations

Three studies, including 13 523 children, classified AKI according to KDIGO stage. The pooled incidences were 7.0%, 3.2% and 0.7% for Stages 1, 2 and 3, respectively (Table 3), with certainty levels rated very low.

#### 3.5.3 AKI‐Incidence by ASA Class

Two studies involving mixed pediatric surgical populations (13 415 children) reported AKI incidence stratified by the ASA classification [32, 35]. The pooled incidence increased with higher ASA class, from 2.4% in ASA 1–2 to 7.5% in ASA 3 and 18.9% in ASA 4–5. In logistic regression adjusted for study, higher ASA class was significantly associated with increased AKI risk (*p* < 0.001 overall; Table 4), with pooled odds ratios of 2.91 (95% CI: 2.40–3.53) for ASA 3 and 7.00 (5.47–8.95) for ASA 4–5 compared with ASA 1–2.

**TABLE 4:** AKI incidence by ASA class.

	No. of studies	No. of individuals	*I* ^2^	Incidence (%)	Odds ratio	*p*	Certainty GRADE
ASA 1–2	2	5752	98.7	2.4 (0.5–11.3)	Ref	—	
ASA 3	2	6861	99.4	7.5 (2.2–22.1)	2.91 (2.40–3.53)	< 0.001	⨁⨁⨁◯ Moderate^a,b^
ASA 4–5	2	802	93.8	18.9 (10.2–32.5)	7.00 (5.47–8.95)	< 0.001	⨁⨁⨁⨁ High^a,b^

*Note:* Incidence of acute kidney injury (AKI) by American Society of Anesthesiologists (ASA) physical status. Odds ratios (ORs) with 95% confidence intervals (CIs) are reported using ASA I–II as the reference. Certainty of evidence was downgraded due to high heterogeneity (a) andimprecision (b). Only adding. (a) and (b) associated with their significance.

#### 3.5.4 AKI‐Incidence by Age Group

The same two studies, including 13 430 children from mixed pediatric surgical populations, also reported AKI incidence stratified by age group [25, 32]. Incidence was higher in younger children, ranging from 9.8% in those aged 0–2 years to 4.0% in adolescents aged 12–18 years. The odds of AKI decreased progressively with increasing age compared with the 12‐ to 18‐year reference group (Table 5), with odds ratios of 1.51 (95% CI, 1.23–1.86; *p* < 0.001) for ages 6–12 years, 2.03 (95% CI, 1.66–2.49; *p* < 0.001) for ages 2–6 years and 2.47 (95% CI, 2.02–3.02; *p* < 0.001) for ages 0–2 years.

**TABLE 5:** AKI‐incidence by age group.

	No. of studies	No. of individuals	*I* ^2^	Incidence (%)	Odds ratio	*p*	Certainty GRADE
Age 0–2	2	2269	98.8	9.8 (2.8–29.1)	2.47 (2.02–3.02)	< 0.001	⨁⨁⨁◯ Moderate^a,b^
Age 2–6	2	2485	99.2	7.6 (1.3–33.9)	2.03 (1.66–2.49)	< 0.001	⨁⨁⨁◯ Moderate^a,b^
Age 6–12	2	3134	98.8	5.8 (1.4–21.0)	1.51 (1.23–1.86)	< 0.001	⨁⨁◯◯ Low^a,b^
Age 12–18	2	5542	97.4	4.0 (1.8–8.9)	Ref	—	

*Note:* Incidence of AKI by age group. Odds ratios (ORs) with 95% confidence intervals (CIs) and *p*‐values from a logistic regression model assessing the association between age and AKI risk. 12–18 years serves as the reference category. Certainty of evidence was downgraded due to high heterogeneity (a) and imprecision (b). Only adding. (a) and (b) associated with their significance.

### 3.6 Reporting Biases

The funnel plot for the overall population is presented in Figure 4, with population‐specific plots (Figure S2) and formal tests of asymmetry (Table S3) provided in the Supporting Information. Formal tests of asymmetry (Peters' and Egger's tests) supported the visual impression of funnel plot asymmetry, suggesting potential small‐study effects and underrepresentation of healthier, lower‐risk populations in the published literature.

**FIGURE 4:**
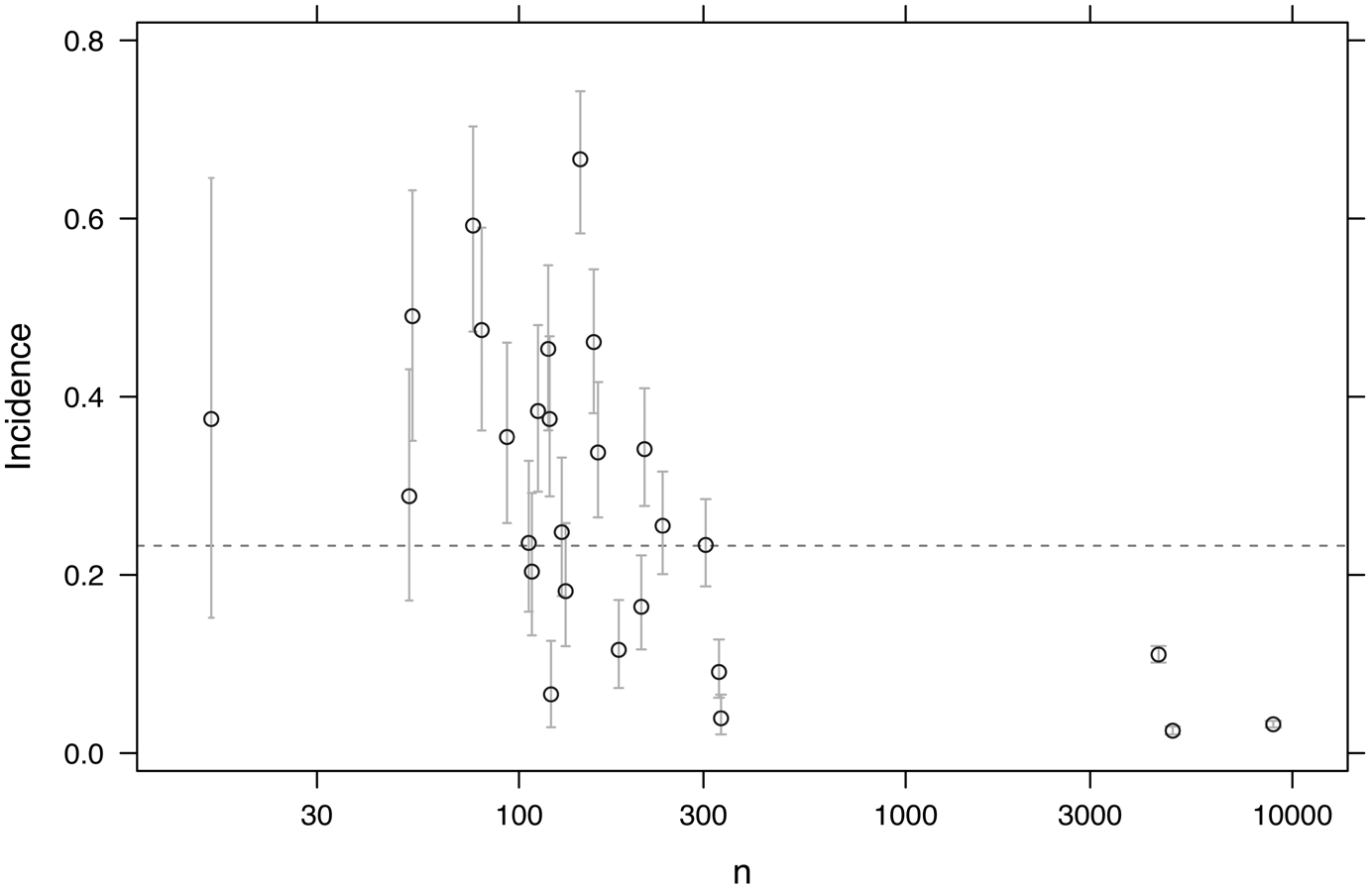
Incidence rates with 95% confidence intervals are plotted against study size. The weighted incidence using a random effects model (0.233) is indicated by a dashed line. Each dot represents an individual study.

Since the KDIGO classification requires a preoperative creatinine measurement, children without such testing, typically representing lower‐risk patients, were systematically excluded, introducing a further selection bias.

Given the considerable heterogeneity, no sensitivity analysis was performed for the overall population. Risk‐of‐bias assessments were also influenced by missing data, as patients without preoperative creatinine values were excluded from KDIGO classification.

### 3.7 Quality of Evidence

For incidence outcomes, the certainty of evidence was rated low to very low across the overall population, surgical subgroups and KDIGO stages, mainly due to retrospective observational design, risk of bias, inconsistency and imprecision (Table S2).

## 4 Discussion

This systematic review and meta‐analysis assessed the literature on the incidence of pediatric postoperative KDIGO‐defined AKI following noncardiac surgery. Twenty‐seven studies published between 2017 and 2024 were analyzed, comprising a total of 21 908 patients. To the best of our knowledge, this is the first meta‐analysis to quantify AKI in this population.

Although the pooled incidence of pediatric postoperative AKI was 23.3%, this estimate should be interpreted with caution. The extreme heterogeneity across studies complicates interpretation of any single pooled estimate. While the use of a random‐effects model was methodologically appropriate given the substantial heterogeneity, the higher incidence observed likely reflects the disproportionate influence of smaller, high‐risk cohorts. Consequently, the pooled estimate is unlikely to accurately represent the broader pediatric surgical population.

The publication bias analysis suggests that low‐risk populations may be underrepresented, potentially leading to an overestimation of AKI incidence. In addition, the restriction to studies applying KDIGO criteria requiring preoperative creatinine measurements likely selected for higher‐risk populations. Children who do not undergo routine preoperative blood testing generally represent a healthier surgical cohort. Consequently, the true incidence of postoperative AKI in the broader pediatric surgical population is likely lower than the rates reported here, which limits the generalizability of these findings.

Rather than relying on a pooled incidence estimate across heterogeneous surgical groups, we judged that population‐specific analyses would better reflect the clinical reality and provide more meaningful estimates of AKI risk for the specific pediatric surgical patient. Accordingly, we analyzed the included studies within three surgical populations: neonatal surgery, liver transplantation and mixed pediatric cohorts spanning all age groups and patient categories.

Our findings showed a pooled incidence of postoperative AKI in neonates of 22.3%, with Stage 1 AKI being the most common. Most included neonates were born after 32 weeks of gestation, and the only study enrolling a younger cohort reported a markedly higher incidence of postoperative AKI. A recent systematic review and meta‐analysis reported an incidence of postoperative AKI of 33% in neonates after noncardiac surgery, with affected patients demonstrating a higher risk of postoperative mortality [48].

The pooled incidence of postoperative AKI following pediatric liver transplantation was 30.1%. Our findings align with previously reported incidence rates and highlight the importance of identifying and applying perioperative renoprotective strategies in this population [49]. Biliary atresia, followed by inborn errors of metabolism, was the most common indication for liver transplantation according to included studies. However, the risk of postoperative AKI in the pediatric liver transplantation cohort is likely to be heavily linked to the pre‐ and peri‐operative patient conditions and influenced by, for example, disease severity, associated syndromes and toxic accumulation of metabolites [50, 51].

The pooled incidence of postoperative AKI in mixed pediatric surgical populations was 9.2%. However, the five studies representing this cohort included in this meta‐analysis demonstrated substantial variation in reported incidence. Three large observational studies, with patients (*n* = 18 331) undergoing a broad spectrum of pediatric surgical procedures, with gastrointestinal operations being most common, reported an incidence of postoperative AKI of 2.5%, 3.2% and 11.0%, respectively [32, 35, 43]. The proportion of elective operations in these cohorts was 100%, 94.3% and 84.7%. Data on emergency surgery were inconsistently reported, limiting a meaningful analysis of AKI incidence in this subgroup.

The remaining two studies further highlight this heterogeneity: a randomized trial of urological reconstructive surgery reported an incidence of 16.4% [46], while a single‐center general pediatric surgical cohort with a higher proportion of emergency procedures and preoperative sepsis reported 35.5% [24]. Taken together, these data suggest that the pooled incidence of 9.2% likely does not fully reflect the broader pediatric surgical population. High‐risk cohorts may contribute to higher incidence estimates, whereas large observational studies with predominately elective procedures report lower risk for postoperative AKI.

Three studies [24, 32, 35] (a total of 13 523 patients) reported postoperative AKI incidence stratified by KDIGO stage. Pooled incidences were 7.0%, 3.2% and 0.7% for Stages 1, 2 and 3, respectively. A fourth large cohort of 4901 elective procedures in our review similarly reported that most AKI episodes were Stage 1, consistent with a predominance of milder AKI [43]. This demonstrates an inverse relationship between disease severity and incidence, where more advanced stages are less frequent but carry greater clinical significance. Given that KDIGO Stage 1 was the most frequently reported stage, the overall pooled incidence is likely influenced by a predominance of mild AKI, the long‐term clinical relevance of which in children remains uncertain.

Two large studies including approximately 13 500 patients allowed for analyses stratified by both ASA class and age [32, 35]. Higher ASA class was significantly associated with increased AKI risk, indicating that AKI incidence is linked to the patient's preoperative risk profile.

In addition to comorbidity burden, age emerged as an important factor associated with postoperative AKI. An increased incidence was observed with decreasing age. This pattern aligns with previous reports in neonatal populations, where lower gestational age and birth weight have been linked to higher rates of creatinine‐ and urine output–defined AKI [52]. It remains uncertain whether this reflects a true increased susceptibility to kidney injury in younger children, including infants and neonates, or an overestimation related to the diagnostic criteria themselves. In particular, the higher incidence may partly reflect limitations of current diagnostic markers, with both serum creatinine and urine output being highly challenging to interpret with regard to AKI in this population.

Although organ‐protective measures are relevant for all patients, risk stratification may help identify those at highest risk. Higher AKI incidence was observed in neonatal populations and in children undergoing liver transplantation, suggesting these groups may benefit from enhanced perioperative monitoring. Associations with ASA physical status and age were based on a limited number of studies and should be interpreted with caution, although consistent with established risk factors.

Our findings highlight the need for standardized reporting and further research into perioperative management and early detection of AKI in pediatric surgery. In adults, a recent randomized trial found a major reduction in the incidence of moderate to severe postoperative AKI, using plasma‐derived biomarkers to guide the use of a supportive care bundle to prevent AKI [49]. With more knowledge of both plasma and urinary biomarkers of AKI in children, those findings may offer a framework for risk‐adapted care also in pediatric patients.

### 4.1 Limitations

A major contributor to the high risk‐of‐bias assessments was missing data. Because KDIGO classification requires preoperative creatinine values, patients without such measurements were excluded. As preoperative creatinine is not routinely obtained in otherwise healthy children, but more commonly in those with comorbidities or higher perioperative risk, this likely introduced selection bias which influenced the observed AKI incidence.

In addition, studies differed substantially in creatinine measurement strategies and inclusion criteria, resulting in widely varying proportions of eligible cases. Such nonrandom missing data and variable postoperative monitoring likely contributed to heterogeneity and may have influenced the incidence estimates of postoperative AKI.

Our strict inclusion criteria, restricting eligibility to KDIGO defined AKI within a seven‐day postoperative window, were chosen to improve methodological homogeneity in AKI staging and postoperative assessment windows and to align with KDIGO as the current consensus definition. Consequently, the number of eligible studies was limited, since several otherwise relevant studies used alternative AKI definitions or different postoperative time windows. However, broadening eligibility would likely have increased heterogeneity in outcome definitions and reduced the specificity of the review question. In addition, many studies applying alternative AKI definitions were performed in earlier eras of perioperative care, which may limit their applicability to modern practice and restrict comparisons with older literature.

The mixed pediatric surgical cohort included a small number of patients from the other surgical populations, but the granularity of the original data was insufficient for stratified analyses. Associations with ASA physical status and age should be interpreted with caution, as they are derived from a small number of studies despite the relatively large number of included patients.

Another key limitation is that the evidence base is dominated by retrospective observational studies, with few prospective studies or randomized trials. Most studies were small and single‐center, and many focused on high‐risk populations, which limits the strength and generalizability of the evidence.

## 5 Conclusions

The reported incidence of postoperative AKI in children undergoing noncardiac surgery varies substantially across studies, reflecting considerable heterogeneity in patient populations. While pooled estimates suggest a measurable burden, the high heterogeneity and generally low certainty of evidence limit interpretation of a single summary estimate. High incidence was observed in neonatal populations and in children undergoing liver transplantation. In mixed pediatric cohorts, the overall incidence of postoperative AKI was 9.2%. However, likely selection of higher‐risk patients limits the generalizability of this estimate to the broader pediatric surgical population. Associations with younger age and higher ASA physical status were observed, although these findings were based on a limited number of studies and should be interpreted with caution. Further large, prospective studies are needed to better characterize postoperative AKI in children.

## Author Contributions

*Design of the review*: Arash Emami, Ebba Sivertsson, Michael Marks‐Hultström, Johan Danielsson, Peter Frykholm, Robert Frithiof. *Statistical analysis*: Johan Westerbergh. *Data extraction*: Ebba Sivertsson, Arash Emami, Robert Frithiof. All authors provided feedback on the manuscript and approved the final version.

## Funding

M.M.‐H. received support from the Swedish Heart Lung Foundation (grant nos 20230627 and 20230732). A.E. received support from the Gillbergska foundation.

## Conflicts of Interest

The authors declare no conflicts of interest.

## Supporting information


**Figure S1:** (a) Traffic light plots for ROBINS‐I assessment of observational trials. Risk‐of‐bias assessment for the included studies using the ROBINS‐I tool. Traffic light plot summarizing judgments across the seven ROBINS‐I domains for the 25 non‐randomized studies. (b) Traffic light plots for RoB 2 assessment of randomized trials. Risk‐of‐bias assessment for the included randomized trials using the RoB 2 tool. Traffic light plot summarizing judgments across the five RoB 2 domains for the two trials.
**Figure S2:** Funnel plots of surgical populations. Funnel plot showing incidence rate with 95% confidence intervals plotted against study size. The weighted incidence for each subgroup is shown as a dashed line.
**Table S3:** Peters and Eggers formal tests of asymmetry.

**Table S1:** Reason for exclusion of full text assessed articles.

**Table S2:** GRADE assessment of the certainty of evidence for study outcomes.

**Data S1:** PED PO‐AKI search strategy.

**Data S2:** PRISMA 2020 Checklist.

## Data Availability

All data supporting the findings of this study are available in the published articles included in the review and in the Supporting Information.
