# Supplementary material for: Pediatric Postoperative Acute Kidney Injury After Noncardiac Surgery: A Systematic Review and Meta‐Analysis
Source: Paediatr Anaesth. 2026 Aug 18;36(11):1387–99. doi: 10.1002/pan.70284 (PMC13639225; doi:10.1002/pan.70284)
Supplement: Supplementary file 1 [file PAN-36-1387-s001.docx]

**ESM Table S2. GRADE assessment of the certainty of evidence for study outcomes.**

**Author(s): AE, ES, RF**

**Question:** Incidence of pediatric PO-AKI, systematic review

**Setting:** non cardiac surgery

**Bibliography:**

| **Certainty assessment** | | | | | | | **№ of patients** | | **Effect** | | **Certainty** | **Importance** |
| --- | --- | --- | --- | --- | --- | --- | --- | --- | --- | --- | --- | --- |
| **№ of studies** | **Study design** | **Risk of bias** | **Inconsistency** | **Indirectness** | **Imprecision** | **Other considerations** | **PO-AKI** | **no comparator** | **Relative (95% CI)** | **Absolute (95% CI)** |  |  |
| **Total Weighted incidence** | | | | | | | | | | | | |
| 27 | non-randomised studies | serious^a^ | very serious^b^ | not serious | serious^c^ | none | 1849/21908 (8.4%) |  | not estimable |  | ⨁◯◯◯ Very low^a,b,c^ | NOT IMPORTANT |
| **KDIGO stage 1 Pediatric** | | | | | | | | | | | | |
| 3 | non-randomised studies | serious^a^ | very serious^b^ | not serious | serious^c^ | none | 523/13523 (3.9%) |  | not estimable |  | ⨁◯◯◯ Very low^a,b,c^ | IMPORTANT |
| **KDIGO stage2 Pediatric** | | | | | | | | | | | | |
| 3 | non-randomised studies | serious^a^ | very serious^b^ | not serious | serious^c^ | none | 234/13523 (1.7%) |  | not estimable |  | ⨁◯◯◯ Very low^a,b,c^ | IMPORTANT |
| **KDIGO stage 3 Pediatric** | | | | | | | | | | | | |
| 3 | non-randomised studies | serious^a^ | very serious^b^ | not serious | not serious | none | 63/13523 (0.5%) |  | not estimable |  | ⨁◯◯◯ Very low^a,b^ | IMPORTANT |
| **KDIGO stage 1 Neonatal** | | | | | | | | | | | | |
| 4 | non-randomised studies | not serious | very serious^b^ | not serious | serious^c^ | none | 68/776 (8.8%) |  | not estimable |  | ⨁◯◯◯ Very low^b,c^ | IMPORTANT |
| **KDIGO stage 2 Neonatal** | | | | | | | | | | | | |
| 4 | non-randomised studies | not serious | serious^d^ | not serious | serious^c^ | none | 27/776 (3.5%) |  | not estimable |  | ⨁⨁◯◯ Low^c,d^ | IMPORTANT |
| **KDIGO stage 3 Neonatal** | | | | | | | | | | | | |
| 4 | non-randomised studies | not serious | very serious^b^ | not serious | serious^c^ | none | 35/776 (4.5%) |  | not estimable |  | ⨁◯◯◯ Very low^b,c^ | IMPORTANT |
| **KDIGO stage 1 LT** | | | | | | | | | | | | |
| 12 | non-randomised studies | not serious | serious^d^ | not serious | serious^c^ | none | 229/1426 (16.1%) |  | not estimable |  | ⨁⨁◯◯ Low^c,d^ | IMPORTANT |
| **KDIGO stage 2 LT** | | | | | | | | | | | | |
| 12 | non-randomised studies | not serious | serious^d^ | not serious | serious^c^ | none | 178/1426 (12.5%) |  | not estimable |  | ⨁⨁◯◯ Low^c,d^ | IMPORTANT |
| **KDIGO stage 3 LT** | | | | | | | | | | | | |
| 12 | non-randomised studies | not serious | not serious | not serious | not serious | none | 76/1426 (5.3%) |  | not estimable |  | ⨁⨁⨁⨁ High | IMPORTANT |

**CI:** confidence interval

#### Explanations

a. Selection bias

b. Considerable heterogeneity >89%

c. Wide CI

d. Heterogeneity 70-80%

**Question:** Age compared to Age 0-2 ref for prognostic factor for PO-AKI

| **Certainty assessment** | | | | | | | **№ of patients** | | **Effect** | | **Certainty** | **Importance** |
| --- | --- | --- | --- | --- | --- | --- | --- | --- | --- | --- | --- | --- |
| **№ of studies** | **Study design** | **Risk of bias** | **Inconsistency** | **Indirectness** | **Imprecision** | **Other considerations** | **Age** | **Ref : Age 12-18** | **Relative (95% CI)** | **Absolute (95% CI)** |  |  |
| **Age 0-2** | | | | | | | | | | | | |
| 2 | non-randomised studies | not serious | very serious^a^ | not serious | serious^b^ | strong association dose response gradient | 205/2269 (9.0%) | 214/5542 (3.9%) | **OR 2.47** (2.02 to 3.02) | **52 more per 1 000** (from 36 more to 70 more) | ⨁⨁⨁◯ Moderate^a,b^ | IMPORTANT |
| **Age 2-6** | | | | | | | | | | | | |
| 2 | non-randomised studies | not serious | very serious^a^ | not serious | serious^b^ | strong association dose response gradient | 187/2485 (7.5%) | 214/5542 (3.9%) | **OR 2.03** (1.66 to 2.49) | **37 more per 1 000** (from 24 more to 52 more) | ⨁⨁⨁◯ Moderate^a,b^ | IMPORTANT |
| **Age 6-12** | | | | | | | | | | | | |
| 2 | non-randomised studies | not serious | very serious^a^ | not serious | serious^b^ | dose response gradient | 181/3314 (5.5%) | 214/5542 (3.9%) | **OR 1.51** (1.23 to 1.86) | **19 more per 1 000** (from 8 more to 31 more) | ⨁⨁◯◯ Low^a,b^ | IMPORTANT |

**CI:** confidence interval; **OR:** odds ratio

#### Explanations

a. High heterogeneity, I2 >90%

b. Wide CI

**Question:** Incidence Postoperative AKI surgical populations

**Bibliography:**

| **Certainty assessment** | | | | | | | **№ of patients** | | **Effect** | | **Certainty** | **Importance** |
| --- | --- | --- | --- | --- | --- | --- | --- | --- | --- | --- | --- | --- |
| **№ of studies** | **Study design** | **Risk of bias** | **Inconsistency** | **Indirectness** | **Imprecision** | **Other considerations** | **Incidence Postoperative AKI** | **[comparison]** | **Relative (95% CI)** | **Absolute (95% CI)** |  |  |
| **Neonatal** | | | | | | | | | | | | |
| 5 | non-randomised studies | not serious | very serious^a^ | not serious | serious^b^ | none | 156/829 (18.8%) |  | not estimable |  | ⨁◯◯◯ Very low^a,b^ | IMPORTANT |
| **Liver transplant** | | | | | | | | | | | | |
| 17 | non-randomised studies | not serious | very serious^a^ | not serious | serious^b^ | none | 716/2448 (29.2%) |  | not estimable |  | ⨁◯◯◯ Very low^a,b^ | IMPORTANT |
| **Pediatric noncardiac surgery** | | | | | | | | | | | | |
| 5 | non-randomised studies | serious^c^ | very serious^a^ | not serious | serious^b^ | none | 977/18631 (5.2%) |  | not estimable |  | ⨁◯◯◯ Very low^a,b,c^ | IMPORTANT |

**CI:** confidence interval

#### Explanations

a. High I2

b. Wide CI

c. Selection bias

**Question:** Effect of ASA class compared to ASA 1-2 Ref in PO-AKI

| **Certainty assessment** | | | | | | | **№ of patients** | | **Effect** | | **Certainty** | **Importance** |
| --- | --- | --- | --- | --- | --- | --- | --- | --- | --- | --- | --- | --- |
| **№ of studies** | **Study design** | **Risk of bias** | **Inconsistency** | **Indirectness** | **Imprecision** | **Other considerations** | **Effect of ASA class** | **ASA 1-2 Ref** | **Relative (95% CI)** | **Absolute (95% CI)** |  |  |
| **ASA 3** | | | | | | | | | | | | |
| 2 | non-randomised studies | not serious | very serious^a^ | not serious | serious^b^ | strong association dose response gradient | 500/6861 (7.3%) | 137/5752 (2.4%) | **OR 2.91** (2.40 to 3.53) | **42 more per 1 000** (from 32 more to 55 more) | ⨁⨁⨁◯ Moderate^a,b^ | IMPORTANT |
| **ASA 4-5** | | | | | | | | | | | | |
| 2 | non-randomised studies | not serious | serious^c^ | not serious | serious^b^ | very strong association dose response gradient | 150/802 (18.7%) | 137/5752 (2.4%) | **OR 7.00** (5.47 to 8.95) | **122 more per 1 000** (from 94 more to 155 more) | ⨁⨁⨁⨁ High^a,b^ | IMPORTANT |

**CI:** confidence interval; **OR:** odds ratio

#### Explanations

a. High heterogeneity >90%

b. Wide CI
