# Supplementary material for: Pediatric Postoperative Acute Kidney Injury After Noncardiac Surgery: A Systematic Review and Meta‐Analysis
Source: Paediatr Anaesth. 2026 Aug 18;36(11):1387–99. doi: 10.1002/pan.70284 (PMC13639225; doi:10.1002/pan.70284)
Supplement: Supplementary file 2 [file PAN-36-1387-s002.docx]

**Table S1: Reason for exclusion of full text assessed articles**

**Not KDIGO (n=63)**

| No | Author | Year | Reason for exclusion |
| --- | --- | --- | --- |
| 1. | Fitski, M. et al. | 2024 | Does not specify AKI definition criteria |
| 2. | Shang, S. et al. | 2024 | Does not specify AKI definition criteria |
| 3. | Engen, R. M. et al. | 2024 | pRIFLE |
| 4. | Aaraj, S. et al. | 2024 | Does not specify AKI definition criteria |
| 5. | Qian, M. Y. et al. | 2024 | KDIGO + AKI diagnostic code within 7 days after surgery |
| 6. | Sinha, S. et al. | 2022 | Does not specify AKI definition criteria |
| 7. | ELHoshy, H. S. et al. | 2022 | Does not specify AKI definition criteria |
| 8. | Zhu, Y. et al. | 2018 | Not KDIGO AKI definition |
| 9. | Keenswijk, W. et al. | 2017 | RIFLE |
| 10. | Bai, S. et al. | 2022 | pRIFLE |
| 11. | Wang, H. H. et al. | 2015 | ICD |
| 12. | Morozov, D. et al. | 2018 | Not KDIGO AKI definition, PO-AKI not outcome |
| 13. | Pessano, S. et al. | 2024 | Not KDIGO AKI definition, PO-AKI not outcome |
| 14. | Farhadi, R. et al. | 2021 | RIFLE, NICU |
| 15. | Cabral, F. C. et al | 2015 | RIFLE PICU |
| 16. | Polo, J. C. G. et al. | 2014 | RIFLE |
| 17. | Mohamed, T. H. et al. | 2023 | ICD |
| 18. | Taniguchi, Y. et al. | 2018 | ICD |
| 19. | Schemm. et al | 2023 | ICD |
| 20. | Zimmerman, LA. et al | 2016 | ARF, no AKI definition |
| 21. | Shum,HP. et al | 2015 | Adult and AKIN |
| 22. | Xie,J. et al. | 2015 | ARF, no AKI definition |
| 23. | Ryan,A. et al | 2020 | pRIFLE |
| 24. | ElBaset, MA. et al | 2022 | AKIN |
| 25. | Sinha, S. et al. | 2022 | Not KDIGO AKI definition |
| 26. | Tu, Z. Z. et al. | 2024 | Does not specify AKI definition criteria |
| 27. | Tafuri, A. et al. | 2022 | AKIN |
| 28. | Ţarcă, E. et al. | 2023 | Not KDIGO AKI definition |
| 29. | Suzuki, M. et al. | 2012 | Not KDIGO AKI definition |
| 30. | Ferah, O. et al. | 2019 | AKIN |
| 31. | Chen, J. et al. | 2021 | pRIFLE |
| 32. | Derkay, C. S. et al. | 2013 | Does not specify AKI definition criteria |
| 33. | Stack, M. et al. | 2016 | Not KDIGO AKI definition |
| 34. | Zheng, C. et al. | 2019 | AKIN |
| 35. | Taylor, M. A. et al. | 2019 | Does not specify AKI definition criteria |
| 36. | Park, J. H. et al. | 2017 | AKIN |
| 37. | Malik, A. T. et al. | 2015 | ARF, no AKI definition |
| 38. | Hernandez et al | 2018 | ARF, no AKI definition |
| 39. | ElSheemy,M. et al | 2015 | Not KDIGO PO-AKI definition |
| 40. | Robinson, C. H. et al. | 2024 | ICD |
| 41. | Ramirez-Amoros, C. et al. | 2024 | ICD |
| 42. | Lee, W. Y. et al. | 2024 | RIFLE |
| 43. | Merckling, M. et al. | 2025 | ICD |
| 44. | Xuan, N. M. et al | 2020 | AKIN |
| 45. | Talving, P. et al. | 2013 | RIFLE |
| 46. | Almardini, R. I. et al. | 2017 | Not KDIGO AKI definition |
| 47. | Kotb, S. et al. | 2013 | Not KDIGO AKI definition |
| 48. | Hawkins, J. et al. | 2022 | Not KDIGO AKI definition |
| 49. | Slater, M. B. et al. | 2016 | RIFLE |
| 50. | Lehenbauer, D. G. et al. | 2018 | AKI defined as serum creatinine >1.5mg/dL or diagnosis documented in the medical record |
| 51. | Zwiers, A. J. M. et al. | 2015 | RIFLE |
| 52. | Sinner, B. et al. | 2020 | pRIFLE |
| 53. | Jöbsis, J. J. et al. | 2017 | AKIN |
| 54. | Menger, R. P. et al. | 2017 | Not KDIGO AKI definition |
| 55. | User İ, R. et al. | 2022 | Does not specify ARF definition criteria |
| 56. | Tøndel, C. et al. | 2012 | Diagnosis based on biopsies, not KDIGO |
| 57. | Kowalewski, G. et al. | 2021 | Not KDIGO AKI definition, PO-AKI not outcome |
| 58. | Ferah, O. et al. | 2019 | AKIN and RIFLE |
| 59. | Yamada, A. et al. | 2014 | Not KDIGO AKI definition, PO-AKI not outcome |
|  | Eklund, J. E. et al. | 2015 | ICD |
| 60. | Jeong, H | 2024 | AKIN |
| 61. | Yang,Y. | 2024 | Not KDIGO AKI definition |
| 62. | Lee, J., | 2019 | AKIN |

**Wrong Outcome (n=96)**

| 1. | McIntire, C. et al. | 2024 | The exposure is the antibiotics, not PO-AKI |
| --- | --- | --- | --- |
| 2. | Lembo, C. et al. | 2024 | The exposure is contrast, not PO-AKI |
| 3. | Garg, P. P. et al. | 2024 | Surgical NEC: timing between surgery and AKI not specified/does not meet PO-AKI criteria. |
| 4. | Sarin, Y. K. et al. | 2024 | PO-AKI is not an outcome |
| 5. | McNab, S. et al. | 2014 | PO-AKI is not an outcome |
| 6. | Yoo, B. M. et al. | 2023 | PO-AKI is not an outcome , CRRT outcome |
| 7. | Zou, Z. et al. | 2023 | NGAL study , not PO-AKI as outcome |
| 8. | Hasankhani, E. G. et al. | 2013 | PO-AKI is not an outcome |
| 9. | Gist, K. M. et al. | 2023 | PICU |
| 10. | Xu, X. et al. | 2018 | Not a postoperative cohort |
| 11. | Fang, F. et al. | 2018 | PO-AKI is not an outcome |
| 12. | Alkandari, O. et al. | 2018 | Study initiates 3 months post-transplant |
| 13. | Sun, Y. T. et al. | 2024 | PO-AKI is not an outcome , CRRT outcome |
| 14. | Riddick, R. et al. | 2023 | NEC and ROP association, not PO-AKI |
| 15. | Garg, P. M. et al. | 2022 | Surgical NEC: timing between surgery and AKI not specified/does not meet PO-AKI criteria. |
| 16. | Garg, P. M. et al. | 2022 | NICU |
| 17. | Szymczak, M. et al. | 2018 | PO-AKI is not an outcome |
| 18. | Dias, R. et al. | 2016 | PO-AKI is not an outcome |
| 19. | Ersoy, Z. et al. | 2017 | Our definition of PO-AKI is not an outcome |
| 20. | Garg, P. M. et al. | 2023 | Surgical NEC: timing between surgery and AKI not specified/does not meet PO-AKI criteria. |
| 21. | Garg, P. M. et al. | 2021 | Surgical NEC: timing between surgery and AKI not specified/does not meet PO-AKI criteria. |
| 22. | Ronco, C. et al. | 2014 | CRRT outcome |
| 23. | You, R. et al. | 2021 | Not a postoperative cohort |
| 24. | Sathish, M. et al. | 2021 | PO-AKI is not an outcome |
| 25. | Lizardo, R. E. et al. | 2015 | PO-AKI is not an outcome |
| 26. | Torabi, J. et al. | 2021 | Not a postoperative cohort |
| 27. | Fonseca, E. et al. | 2014 | PO-AKI is not an outcome |
| 28. | Bakkaloğlu, S. A. et al. | 2024 | Crush AKI, not PO-AKI |
| 29. | Santiago, A. K. C. et al. | 2021 | PO-AKI is not an outcome |
| 30. | Telefon, A. H. et al. | 2024 | Crush AKI, not PO-AKI |
| 31. | Akbaba, B. et al. | 2024 | Crush AKI, not PO-AKI |
| 32. | Sun, Y. T. et al. | 2024 | PO-AKI is not an outcome, not clear AKI definition. |
| 33. | Garg, P. M. et al. | 2024 | Surgical NEC: timing between surgery and AKI not specified/does not meet PO-AKI criteria. |
| 34. | Fan, H. et al. | 2024 | PO-AKI is not an outcome |
| 35. | Høyer, S. et al. | 2024 | PO-AKI is not an outcome |
| 36. | Glass, A. et al. | 2025 | Not our PO-AKI definition |
| 37. | Aftzoglou, M. et al. | 2025 | PO-AKI is not an outcome |
| 38. | Kalita, D. et al. | 2023 | NICU |
| 39. | Sutherland, S. M. et al. | 2015 | Not a postoperative cohort |
| 40. | McNicol, E. D. et al. | 2016 | PO-AKI is not an outcome |
| 41. | Li, Z. H. et al. | 2014 | Not a postoperative cohort |
| 42. | Hanson, H. R. et al. | 2018 | ER, Not a postoperative cohort |
| 43. | Suchar, A. M. et al. | 2023 | PO-AKI is not an outcome |
| 44. | Robinson, C. H. et al. | 2021 | Not a postoperative cohort |
| 45. | Garg, P. M. et al. | 2022 | Surgical NEC: timing between surgery and AKI not specified/does not meet PO-AKI criteria. |
| 46. | Garg, P. M. et al. | 2023 | Surgical NEC: timing between surgery and AKI not specified/does not meet PO-AKI criteria. |
| 47. | Garg, P. M. et al. | 2022 | Surgical NEC: timing between surgery and AKI not specified/does not meet PO-AKI criteria. |
| 48. | Garg, P. M. et al. | 2023 | Surgical NEC: timing between surgery and AKI not specified/does not meet PO-AKI criteria. |
| 49. | Kim, M. K. et al. | 2023 | Not our PO-AKI definition |
| 50. | GaŁĄzka, P. et al. | 2021 | Unclear definition and timing of surgery; focus on NEC and outcomes rather than PO-AKI. |
| 51. | Fly, J. H. et al. | 2024 | Focus on severe NEC, antibiotics and AKI; not PO-AKI |
| 52. | Leonard, J. C. et al. | 2021 | Bleeding compliacations, not PO-AKI |
| 53. | Kuok et al | 2022 | Crush AKI |
| 54. | D'Arienzo, D et al | 2019 | PICU |
| 55. | AlGadeeb et al | 2021 | NICU |
| 56. | Charlton, J. R. et al. | 2020 | NICU |
| 57. | Youssef, D. et al. | 2019 | NICU |
| 58. | Jetton, J. G. et al. | 2022 | NICU |
| 59. | Ayhan, A. et al. | 2017 | NICU |
| 60. | Charlton, J. R. et al. | 2019 | CRRT outcomes ,not PO-AKI |
| 61. | Mahgoob, M. H. et al. | 2023 | NICU |
| 62. | Zozaya, C. et al. | 2020 | NICU |
| 63. | Sun, Y. et al. | 2022 | Not our PO-AKI definition |
| 64. | Han, R. et al. | 2023 | NICU population |
| 65. | Silver, L. J. et al. | 2022 | PICU |
| 66. | Criss, C. N. et al. | 2018 | NICU |
| 67. | Chanchlani, R. et al. | 2019 | PICU |
| 68. | Klib, M. et al. | 2023 | Not a postoperative cohort |
| 69. | Raina, R. et al. | 2024 | NICU |
| 70. | Volpon, L. C. et al. | 2016 | PICU |
| 71. | Ferreira, M. C. D. et al. | 2020 | PICU |
| 72. | Hessey, E. et al. | 2020 | PICU |
| 73. | Kriplani, D. S. et al. | 2016 | PICU |
| 74. | Krishnasamy, S. et al. | 2025 | PICU |
| 75. | Chisavu, F. et al. | 2024 | PICU |
| 76. | Zhang, X. et al. | 2024 | PICU |
| 77. | Ladeiras, R. et al. | 2019 | NICU |
| 78. | Palermo, J. et al. | 2017 | PICU |
| 79. | Raman, S. et al. | 2020 | PICU |
| 80. | Ulrich, E. H. et al. | 2024 | PICU |
| 81. | Ramya, K. et al. | 2024 | NICU |
| 82. | Alves, M. T. B. et al. | 2024 | PICU |
| 83. | Hessey, E. et al. | 2017 | PICU |
| 84. | Ninmer, E. K. et al. | 2022 | PICU |
| 85. | Rutledge, A. D. et al. | 2024 | NICU |
| 86. | Ghazi, P. et al. | 2014 | Not a postoperative cohort |
| 87. | Garg, P. M. et al. | 2024 | Surgical NEC: timing between surgery and AKI not specified/does not meet PO-AKI criteria. |
| 88. | Goddard, G. R. et al. | 2022 | Outcomes reported KDIGO stage at isolated time points (POD 1, 3, 5); not cumulative PO-AKI, data not comparable |
| 89. | Liberio, B. M. | 2021 | AKI after 30 days postoperatively |
| 90. | Luglio, M. et al. | 2023 | PO-AKI until 14 days after operation |
| 91. | Silva, S. C et al | 2024 | PO-AKI is not an outcome |
| 92. | Raina,R et al | 2024 | Not a postoperative cohort |
| 93. | Chae,H.H et al | 2024 | PO-AKI is not an outcome |
| 94. | Sethi, S. K. et al. | 2022 | NICU |
| 95. | Hui, W. F. et al. | 2023 | PICU |
| 96. | Askenazi, D. J. | 2020 | NICU |

**Wrong publication type (n=28)**

| 1. | Schuermans, A. et al. | 2023 | Review |
| --- | --- | --- | --- |
| 2. | Coleman, C. et al. | 2022 | Review |
| 3. | Selewski, D. T. et al. | 2015 | Review |
| 4. | Yang, H. D. et al. | 2017 | Review |
| 5. | Fragasso, T. et al. | 2018 | Review |
| 6. | Sigurjonsdottir, V. et al. | 2018 | Review |
| 7. | Ojo, B. et al. | 2022 | Review |
| 8. | Gist, K. M. et al. | 2022 | Review |
| 9. | Khandelwal, P. et al. | 2022 | Review |
| 10. | Kirpalani, A. et al. | 2021 | Review |
| 11. | Che, R. et al. | 2020 | Review |
| 12. | Xue, F. S. et al. | 2018 | Letter to editor |
| 13. | Lin, Q. et al. | 2018 | Review |
| 14. | Robinson, C. H. et al. | 2023 | Review |
| 15. | Atkinson | 2021 | Review |
| 16. | Zhong, J. | 2023 | Review |
| 17. | Gorga, S. M. et al. | 2023 | Review |
| 18. | Park, S. K. et al. | 2017 | Letter to the editor |
| 19. | Duong, M. D. et al. | 2023 | Review |
| 20. | Klinkner, D. B. et al. | 2019 | Review |
| 21. | Ziesenitz, V. C. et al. | 2022 | Review |
| 22. | Garg, P. M. et al. | 2024 | Review |
| 23. | Sutherland, S. M. | 2023 | Review |
| 24. | Matloff, R. G. et al. | 2015 | Review |
| 25. | Raina, R. et al. | 2023 | Review |
| 26. | Starr, M. C. et al. | 2021 | Review |
| 27. | Chirico, V | 2024 | Review |
| 28. | Mohidin, B | 2025 | Review |

**Other reasons (n=32)**

| 1. | Hu,J et al | 2024 | Cardiac surgery |
| --- | --- | --- | --- |
| 2. | Wang, B. et al. | 2024 | Cardiac surgery |
| 3. | Onal et al | 2022 | Cardiac surgery |
| 4. | Christensen, A. L. et al. | 2021 | Adult |
| 5. | Bulger, E. M. et al. | 2019 | Adult |
| 6. | Ounissi, M. et al. | 2012 | Adult |
| 7. | Lee, J. et al. | 2013 | Adult |
| 8. | Shankar, S. et al. | 2021 | Adult |
| 9. | Ahmed, N. et al. | 2020 | Adult |
| 10. | Anghelescu, D. L. et al. | 2019 | Adult |
| 11. | Wu, H. C. et al. | 2015 | Adult |
| 12. | Esezobor, C. I | 2024 | Adult |
| 13. | Umar S et al | 2022 | Protocol |
| 14. | Matthias Görges | 2015 | Protocol |
| 15. | Lingappan, K. et al. | 2023 | Protocol |
| 16. | Oudmaijer, C. A. J. et al. | 2022 | Protocol |
| 17. | Bhatt, G. C. et al. | 2022 | Protocol |
| 18. | Elsheemy | 2014 | Protocol |
| 19. | Estefanía, K. | 2023 | no full text |
| 20. | Anjum, S. et al. | 2019 | no full text |
| 21. | Liu,Z.L et al | 2016 | no full text |
| 22. | Friedman, S. et al. | 2015 | no full text |
| 23. | Tang et al | 2018 | no full text |
| 24. | Tan, X. Q. et al. | 2020 | no full text |
| 25. | Sahinturk, H. et al. | 2018 | Same cohort |
| 26. | Sahinturk, H. et al. | 2021 | Same cohort |
| 27. | Garg, P. M. et al. | 2024 | Same cohort |
| 28. | Cui, Y. et al. | 2020 | Same Cohort |
| 29. | Demiröz, D. et al. | 2024 | same cohort |
| 30. | Oudmaijer, C. A. J. et al. | 2021 | Duplicate |
| 31. | Kitai, Y. et al. | 2025 | wrong population, Mice |
| 32. | Zhang, Y. et al. | 2020 | corrigendum |
