# Supplementary material for: Pediatric Postoperative Acute Kidney Injury After Noncardiac Surgery: A Systematic Review and Meta‐Analysis
Source: Paediatr Anaesth. 2026 Aug 18;36(11):1387–99. doi: 10.1002/pan.70284 (PMC13639225; doi:10.1002/pan.70284)
Supplement: Supplementary file 3 [file PAN-36-1387-s003.docx]

| PubMed, 2025-02-14 | | | |
| --- | --- | --- | --- |
| Search no | Search terms | Results | Comments |
| 1 | Acute Kidney Injury[MeSH] | 57 637 |  |
| 2 | "acute kidney damage" OR "acute kidney dysfunction" OR "Acute Kidney Injur*" OR "Acute Kidney Insufficienc*" OR "Acute Kidney Failure*" OR "Acute Kidney Tubular Necrosis" OR "acute renal damage" OR "acute renal dysfunction" OR "Acute Renal Injur*" OR "Acute Renal Insufficienc*" OR "Acute Renal Failur*" OR "acute tubular damage" OR "acute tubular injur*" OR "acute tubular necrosis" OR AKI OR ARF OR ATN [Title/Abstract] | 83 337 |  |
| 3 | 1 OR 2 | 100 621 |  |
| 4 | "Perioperative Period"[Mesh:NoExp] OR "Postoperative Period"[Mesh] OR "Postoperative Complications"[Mesh] | 674 502 |  |
| 5 | perioperative* OR postoperative* OR surg* [Title/Abstract] | 2 734 817 |  |
| 6 | 4 OR 5 | 2 991 360 |  |
| 7 | "Child"[Mesh] OR "Child Health"[Mesh] OR "Infant"[Mesh] OR "Adolescent"[Mesh] OR "Adolescent Health"[Mesh] OR "Pediatrics"[Mesh] | 4 046 094 |  |
| 8 | adolescen* OR babies OR baby OR child* OR infanc* OR infant* OR newborn* OR new-born* OR neonat* OR paediatric* OR pediatric* OR prematur* OR preterm OR puberty OR teen* OR toddler* OR youth [Title/Abstract] | 2 948 860 |  |
| 9 | 7 OR 8 | 4 962 165 |  |
| 10 | 3 AND 6 AND 9 | 2 494 |  |
| 11 | 2024/01/01:3000/12/31[Date - Publication] | 196 852 |  |
|  | 10 AND 11 | 180 |  |

| Web of Science, 2025-02-14 | | | |
| --- | --- | --- | --- |
| Search no | Search terms | Results | Comments |
| 1 | "acute kidney damage" OR "acute kidney dysfunction" OR "Acute Kidney Injur*" OR "Acute Kidney Insufficienc*" OR "Acute Kidney Failure*" OR "Acute Kidney Tubular Necrosis" OR "acute renal damage" OR "acute renal dysfunction" OR "Acute Renal Injur*" OR "Acute Renal Insufficienc*" OR "Acute Renal Failur*" OR "acute tubular damage" OR "acute tubular injur*" OR "acute tubular necrosis" OR AKI OR ARF OR ATN [Topic] | 115 332 |  |
| 2 | perioperative* OR postoperative* OR surg* [Topic] | 2 852 170 |  |
| 3 | adolescen* OR babies OR baby OR child* OR infanc* OR infant* OR newborn* OR new-born* OR neonat* OR paediatric* OR pediatric* OR prematur* OR preterm OR puberty OR teen* OR toddler* OR youth [Topic] | 4 291 715 |  |
| 4 | 3 AND 6 AND 9 | 2 005 |  |
| 5 | Filter: 2024-01-01 : 2025-02-14 | 159 |  |

| Cochrane, 2025-02-14 | | | |
| --- | --- | --- | --- |
| Search no | Search terms | Results | Comments |
| 1 | Acute Kidney Injury[MeSH] | 2 472 |  |
| 2 | "acute kidney damage" OR "acute kidney dysfunction" OR  OR "Acute Kidney Tubular Necrosis" OR "acute renal damage" OR "acute renal dysfunction"  OR "acute tubular damage"  OR "acute tubular necrosis" OR AKI OR ARF OR ATN | 3984 |  |
| 3 | Acute NEXT Kidney NEXT Injur* | 5759 |  |
| 4 | Acute NEXT Kidney NEXT Insufficienc* | 19 |  |
| 5 | Acute NEXT Kidney NEXT Failure* | 3376 |  |
| 6 | Acute NEXT Renal NEXT Injur* | 190 |  |
| 7 | Acute NEXT Renal NEXT Insufficienc* | 63 |  |
| 8 | acute NEXT tubular NEXT injur* | 16 |  |
| 9 | #2 OR #3 OR #4 OR #5 OR #6 OR #7 OR #8 | 8470 |  |
| 10 | #1 OR #9 | 8485 |  |
| 11 | "Perioperative Period"[Mesh:NoExp] | 384 |  |
| 12 | "Postoperative Period"[Mesh] | 7625 |  |
| 13 | "Postoperative Complications"[Mesh] | 56840 |  |
| 14 | #11 OR #12 OR #13 | 62497 |  |
| 15 | perioperative* OR postoperative* OR surg* | 423476 |  |
| 16 | #14 OR #15 | 425258 |  |
| 17 | "Child"[Mesh] | 83857 |  |
| 18 | "Child Health"[Mesh] | 283 |  |
| 19 | Infant"[Mesh] | 47086 |  |
| 20 | "Adolescent"[Mesh] | 139606 |  |
| 21 | "Adolescent Health"[Mesh] | 75 |  |
| 22 | "Pediatrics"[Mesh] | 1060 |  |
| 23 | #17 OR #18 OR #19 OR #20 OR #21 OR #22 | 209451 |  |
| 24 | adolescen* OR babies OR baby OR child* OR infanc* OR infant* OR newborn* OR new-born* OR neonat* OR paediatric* OR pediatric* OR prematur* OR preterm OR puberty OR teen* OR toddler* OR youth [Title/Abstract] | 420878 |  |
| 9 | #23 OR #24 | 420891 |  |
| 10 | #10 AND #16 AND #25 | 430 |  |
|  | with Cochrane Library publication date from Jan 2024 to Mar 2025 | 46 |  |

# Sökning PubMed 240306

| Söknr | Söktermer | Antal referenser | Kommentar |
| --- | --- | --- | --- |
|  | Njursvikt |  |  |
| 1 | Acute Kidney Injury[MeSH] | 57 637 |  |
| 2 | "acute kidney damage" OR "acute kidney dysfunction" OR "Acute Kidney Injur*" OR "Acute Kidney Insufficienc*" OR "Acute Kidney Failure*" OR "Acute Kidney Tubular Necrosis" OR "acute renal damage" OR "acute renal dysfunction" OR "Acute Renal Injur*" OR "Acute Renal Insufficienc*" OR "Acute Renal Failur*" OR "acute tubular damage" OR "acute tubular injur*" OR "acute tubular necrosis" OR AKI OR ARF OR ATN [Title/Abstract] | 83 337 |  |
| 3 | 1 OR 2 | 100 621 |  |
|  | Efter operation |  |  |
| 4 | "Perioperative Period"[Mesh:NoExp] OR "Postoperative Period"[Mesh] OR "Postoperative Complications"[Mesh] | 674 502 |  |
| 5 | perioperative* OR postoperative* OR surg* [Title/Abstract] | 2 734 817 |  |
| 6 | 4 OR 5 | 2 991 360 |  |
|  | Barn |  |  |
| 7 | "Child"[Mesh] OR "Child Health"[Mesh] OR "Infant"[Mesh] OR "Adolescent"[Mesh] OR "Adolescent Health"[Mesh] OR "Pediatrics"[Mesh] | 4 046 094 |  |
| 8 | adolescen* OR babies OR baby OR child* OR infanc* OR infant* OR newborn* OR new-born* OR neonat* OR paediatric* OR pediatric* OR prematur* OR preterm OR puberty OR teen* OR toddler* OR youth [Title/Abstract] | 2 948 860 |  |
| 9 | 7 OR 8 | 4 962 165 |  |
| 10 | 3 AND 6 AND 9 | 2 494  1 283 (2012-2024) |  |

(("acute kidney injury"[MeSH Terms] OR ("acute kidney damage"[Title/Abstract] OR "acute kidney dysfunction"[Title/Abstract] OR "acute kidney injur*"[Title/Abstract] OR "acute kidney insufficienc*"[Title/Abstract] OR "acute kidney failure*"[Title/Abstract] OR "Acute Kidney Tubular Necrosis"[Title/Abstract] OR "acute renal damage"[Title/Abstract] OR "acute renal dysfunction"[Title/Abstract] OR "acute renal injur*"[Title/Abstract] OR "acute renal insufficienc*"[Title/Abstract] OR "acute renal failur*"[Title/Abstract] OR "acute tubular damage"[Title/Abstract] OR "acute tubular injur*"[Title/Abstract] OR "acute tubular necrosis"[Title/Abstract] OR "AKI"[Title/Abstract] OR "ARF"[Title/Abstract] OR "ATN"[Title/Abstract])) AND ("Perioperative Period"[MeSH Terms:noexp] OR "Postoperative Period"[MeSH Terms] OR "Postoperative Complications"[MeSH Terms] OR ("perioperative*"[Title/Abstract] OR "postoperative*"[Title/Abstract] OR "surg*"[Title/Abstract])) AND ("Child"[MeSH Terms] OR "Child Health"[MeSH Terms] OR "Infant"[MeSH Terms] OR "Adolescent"[MeSH Terms] OR "Adolescent Health"[MeSH Terms] OR "Pediatrics"[MeSH Terms] OR ("adolescen*"[Title/Abstract] OR "babies"[Title/Abstract] OR "baby"[Title/Abstract] OR "child*"[Title/Abstract] OR "infanc*"[Title/Abstract] OR "infant*"[Title/Abstract] OR "newborn*"[Title/Abstract] OR "new born*"[Title/Abstract] OR "neonat*"[Title/Abstract] OR "paediatric*"[Title/Abstract] OR "pediatric*"[Title/Abstract] OR "prematur*"[Title/Abstract] OR "preterm"[Title/Abstract] OR "puberty"[Title/Abstract] OR "teen*"[Title/Abstract] OR "toddler*"[Title/Abstract] OR "youth"[Title/Abstract]))) AND (2012:2024[pdat])
