# Supplementary material for: Pediatric Postoperative Acute Kidney Injury After Noncardiac Surgery: A Systematic Review and Meta‐Analysis
Source: Paediatr Anaesth. 2026 Aug 18;36(11):1387–99. doi: 10.1002/pan.70284 (PMC13639225; doi:10.1002/pan.70284)
Supplement: Supplementary file 5 [file PAN-36-1387-s005.docx]

Supplementary figures and tables to:

Pediatric postoperative Acute Kidney Injury after non-cardiac surgery: A systematic review and meta-analysis

Arash Emami^1^*, Ebba Sivertsson^1^, Johan Westerbergh^2^, Michael Marks-Hultström^1,3^, Johan Danielsson^4^, Peter Frykholm^1^, Robert Frithiof^1^

**Figures:**

**S1a Traffic light plots for ROBINS-I assessment of observational trials**

**Figure S1a :**Risk-of-bias assessment for the included studies using the ROBINS-I tool. Traffic light plot summarising judgments across the seven ROBINS-I domains for the 25 non-randomised studies.

**Figure S1b: Traffic light plots for RoB 2 assessment of randomised trials**

**Figure S1b:** Risk-of-bias assessment for the included randomised trials using the RoB 2 tool. Traffic light plot summarising judgments across the five RoB 2 domains for the two trials***.***

**Figure S2: Funnel plots of surgical populations**

Figure S2: Funnel plot showing incidence rate with 95% confidence intervals plotted against study size. The weighted incidence for each subgroup is shown as a dashed line.

**Tables**

**Table S1: Reason for exclusion of full text assessed articles**

See separate document.

**Table S2:** GRADE assessment of the certainty of evidence for study outcomes.

See separate document

**Table S3: Peters and Eggers formal tests of asymmetry**

| Test | Estimate | P |
| --- | --- | --- |
| Peters test | 59.31 | 0.045 |
| Eggers test | -3.07 | 0.007 |
